# Effect of pulp capping materials on odontogenic differentiation of human dental pulp stem cells: An in vitro study

**DOI:** 10.1002/cre2.816

**Published:** 2023-12-06

**Authors:** Mahmoud M. Bakr, Mohamed Shamel, Shereen N. Raafat, Robert M. Love, Mahmoud M. Al‐Ankily

**Affiliations:** ^1^ School of Medicine and Dentistry Griffith University Gold Coast Queensland Australia; ^2^ Oral Biology Department, Faulty of Dentistry The British University in Egypt Cairo Egypt; ^3^ Department of Pharmacology and Toxicology, Faculty of Dentistry The British University in Egypt Cairo Egypt

**Keywords:** dental pulp regeneration, odontogenic differentiation, pulp capping, stem cells

## Abstract

**Objectives:**

Migration and differentiation of human dental pulp stem cells (hDPSCs) is a vital and key factor in the success of reparative dentin formation for maintenance of pulp vitality. Pulp capping materials are used to stimulate DPSCs to induce new dentin formation. Thus, the aim of the present study was to compare the response of DPSCs to four commercially available pulp capping materials: a bioactive bioceramic (Material 1), a nonresinous ready‐to‐use bioceramic cement (Material 2), a bioactive composite (Material 3), and a biocompatible, dual‐cured, resin‐modified calcium silicate (Material 4).

**Materials and Methods:**

hDPSCs were isolated and cultured from freshly extracted teeth and were then characterized by flow cytometry and multilineage differentiation. Discs prepared from pulp capping materials were tested with hDPSCs and MTT (3‐(4,5‐dimethylthiazol‐2‐yl)‐2,5‐diphenyltetrazolium bromide) assay, cell migration assay and odontogenic differentiation assay was performed. Expression of osteogenic markers (osteopontin, RUNX family transcription factor 2, osteocalcin) and the odontogenic marker (dentin sialophosphoprotein) was detected using reverse transcription‐polymerase chain reaction.

**Results:**

Materials 1, 2, and 3 generated more cell viability than Material 4. Furthermore, Material 4 showed the least wound exposure percentage, while Material 3 showed the highest percentage. Enhanced mineralization was found in hDSCPs cultured with Material 3, followed by Material 1, and then Material 2, while Material 4 revealed the least calcified mineralization.

**Conclusions:**

The results of this study were inconclusive regards contemporary bioceramic materials designed for vital pulp therapy as they have different effects on hDPSC. Further testing for cytotoxicity using live‐dead staining, animal experiments, clinical trials, and independent analyses of these biomaterials is necessary for clinicians to make an informed decision for their use.

## 1 INTRODUCTION

Maintenance of pulp vitality in restorative procedures has become increasingly feasible due to emerging advanced treatments (Nakashima & Akamine, 2005; Qureshi et al., 2014; Sharaan et al., 2021). Direct pulp capping is a vital pulp therapy for a pin‐point dental pulp exposure when there is no inflammation (Hanna et al., 2020; Kim et al., 2021; Pathak, 2017; Qureshi et al., 2014). Applying a pulp capping material leads to the formation of a dentin bridge and protects pulp vitality through the stimulation and differentiation of the dental pulp stem cells (Pathak, 2017). This technique utilizes a bioactive material that is placed directly over the exposed dental pulp tissue. Pulp capping materials function to prevent a connection between the dental pulp and oral cavity, thus reducing potential inflammation and bacterial infection. At the same time, the used material stimulates dental pulp healing by inducing new dentin termed reparative or tertiary dentin (Wells et al., 2019).

For the protective mechanism of the pulp capping material, dental pulp cells must generally proliferate and migrate to the injured site and then subsequently differentiate into odontoblast‐like cells that form tertiary dentin that distances the pulp from the cavity (Goldberg, 2011). Consequently, human dental pulp stem cells (hDPSCs) have been reported as a critical key for reparative dentin formation (Huang et al., 2021; Nakashima & Akamine, 2005). HDPSCs express mesenchymal stem cell (MSC) markers and have multipotential differentiation ability (Huang et al., 2009; Khampatee et al., 2022; Louwakul et al., 2021; Yaemkleebbua et al., 2019). It has been shown that hDPSCs can differentiate into ectodermal‐, mesodermal‐, and endodermal‐derived cells (Nakashima & Akamine, 2005; Xiao & Nasu, 2014). Hence, these cells are proposed as an alternative cell source for various regenerative applications (Manaspon et al., 2021; Xiao & Nasu, 2014).

An ideal pulp capping material should prevent bacterial infiltration, trigger minimal inflammation, and induce dentin bridge formation (Qureshi et al., 2014). Current materials clinically used for pulp capping can be generally divided into calcium hydroxide (Ca(OH)_2_) (Peng et al., 2022; Whitehouse et al., 2021; Youssef et al., 2019), mineral trioxide aggregate (MTA) (Babaki et al., 2020; Youssef et al., 2019), calcium silicate (Kang, 2020; Kim et al., 2021; Onay et al. 2018), and adhesive‐based materials (Giraud et al., 2019). Interestingly, MTA and calcium silicate‐based materials have a comparable effect on dentin bridge formation, inflammatory response, and success rate (Babaki et al., 2020; Paula et al., 2018). Apart from these clinical observations, direct comparison of the effects of these materials on hDPSCs in vitro is limited. Thus, the aim of the present study was to compare the response of hDPSCs to commercially available materials for vital pulp therapy. The effects of these materials on cell proliferation and odonto/osteogenic differentiation were examined.

## 2 MATERIALS AND METHODS

### 2.1 Isolation and culture of hDPSCs

Freshly extracted teeth, extracted for orthodontic treatment, were collected from human adult patients (18–25 years old) from the dental hospital of the Faculty of Dentistry, The British University in Egypt. Dental pulp tissues were separated from the teeth and cell isolation was performed by tissue explantation. The cells were maintained in Dulbecco's modified Eagle's medium (DMEM) supplemented with 10% fetal bovine serum (FBS) (Gibco), 1% l‐glutamine, 100 U/mL penicillin, and 100 μg/mL streptomycin (Gibco).

The isolated dental pulps were cut into small pieces and digested in a solution of 3 mg/mL type I collagenase (Sigma‐Aldrich) for 3 h at 37°C in order to separate cells. Subsequently, the solution was ﬁltered through a 70‐mm cell strainer (Becton/Dickinson). The single‐cell suspensions were seeded in 35‐mm culture dishes and maintained in a medium containing DMEM supplemented with 15% FBS (Life Technologies) and 1% penicillin–streptomycin (10,000 U/mL) (Life Technologies) as antibiotics. Cells were incubated at 37°C in a 95% humidified atmosphere and 5% CO_2_. The medium was changed every 3 days until cells reached 80% confluency. Passaging of the cells was done using trypsin‐EDTA every 5–7 days. Cells of the fourth passage were used in this study.

### 2.2 Characterization of the hDPSCs

The cultured cells were identified by characterization of the hDPSCs by ﬂow cytometry analysis and multiple lineage differentiation potential.

#### 2.2.1 Flow cytometry

Cells were harvested using trypsin/EDTA solution to obtain a single‐cell suspension. The cells were immunostained in 1% horse serum (Gibco) in sterile phosphate‐buffered saline (PBS) with primary antibodies conjugated with fluorescent dye. Cells were trypsinized and incubated in PBS containing 0.1% FBS for 45 min with fluorescein‐conjugated monoclonal antibodies against CD34, CD44, CD45, CD19, CD73, CD90, and CD105 (BD Biosciences). The flow cytometry test was performed using a flow cytometer (FACSCalibur; BD Biosciences).

#### 2.2.2 Multilineage differentiation

Multilineage differentiation of cultured hDPSCs was performed to detect adipogenic, chondrogenic, and osteogenic differentiation ability (Zhang et al., 2016).

### 2.3 Pulp capping materials disc preparation

Four different pulp capping materials were used in this study: Neoputty‐ MTA® (Avalon Biomed), Bio‐C sealer® (Angelus), Theracal PT® (Bisco), and Activa Bioactive® (BA, Pulpdent). The materials were mixed according to the manufacturer's instructions. Discs of each pulp capping material were shaped under aseptic conditions in 6‐well plates (JET BIOFIL) 35 mm in diameter and 2 mm high, sterilized using ultraviolet irradiation for 15 min, and stored in an incubator (Binder GmbH) at 37°C for 48 h to achieve a complete setting. To prepare material extracts (conditioned medium), the proposed materials were stored in DMEM (Life Technologies) for 24 h at 37°C in a 95% humidified atmosphere and 5% CO_2_. In accordance with the guidelines of the International Organization for Standardization 10993‐5, the ratio of material surface area to medium volume was set at approximately 1.5 cm^2^/mL. The extraction medium was filtered with sterile filters of 0.22 µm pore size (Sartorius A. G. Goettingen).

### 2.4 Cell viability (MTT assay)

At 80% confluence, 1 × 10^3^ cells were seeded in polylysine‐coated 96‐well plates (SPL). The cells of all different groups were incubated with MTT (3‐(4,5‐dimethylthiazol‐2‐yl)‐2,5‐diphenyltetrazolium bromide) reagent. Then, the proliferation was assessed (the number of proliferated viable cells) using the absorbance quantitative enzyme‐linked immunosorbent assay technique at 450 nm wavelength at Days 1, 3, and 7 (Kamiloglu et al., 2020; Alhazmi et al., 2023).

### 2.5 Cell migration assay

Cell migration was performed using an in vitro scratch assay. hDPSCs at a concentration of 2 × 10^5^ cells/well were seeded into 24‐well plates and maintained in a normal growth medium for 24 h. The culture medium was then replaced with a serum‐free culture medium and cultured for 24 h. A scratch was created using a sterilized pipette tip and the cells were exposed to 25% of each material's extracted medium. Images were captured using an inverted phase‐contrast microscope at the initial time, Days 2 and 3 at the same location. Wound closures were calculated from at least three images from the same frame as the initial time image (Suarez‐Arnedo et al., 2020).

### 2.6 In vitro differentiation assay

hDPSCs were cocultured with various material extracts and serum‐free α‐minimum essential medium (α‐MEM). The normal culture medium containing α‐MEM supplemented with 10% FBS was used as a blank control medium. Cells cultured with osteogenic differentiation medium were used as a positive control. Media consisted of the aforementioned normal culture medium supplemented with 50 mg/mL ascorbic acid, 10 mmol/L β‐glycerophosphate, and 10 nmol/L dexamethasone (Sigma).

#### 2.6.1 Alizarin Red staining and quantification

Approximately 1 × 10^5^ hDPSCs were cultured with the elutes supplemented with serum‐free α‐MEM in 6‐well plates for 2 weeks. The cells were washed with PBS and fixed with dehydrated ethanol for 20 min. Cells were stained using the Alizarin Red S Kit (Leagene Biotechnology) according to the manufacturer's instructions for 20 min and then washed with H_2_O five times. Qualitative and quantitative analysis of Alizarin Red staining was performed after the cells were imaged using an inverted light microscope, and the percentage of the area from the mineralization was determined using the ImageJ software.

#### 2.6.2 Alkaline phosphatase activity assay

In a 24‐well plate, monolayers of hDPSC were washed twice with PBS and then once with 0.5 mL alkaline phosphatase buffer. A measure of 250 µL of bone‐specific alkphase B was added to each well and an equal volume of *p*‐nitrophenyl phosphate, disodium salt equilibrated to 4°C was added. Immediately 50 µL was removed and mixed with an aliquot of NaOH to stop the reaction in each well. The previous step was repeated every minute for 10 min. The rate of accumulation of *p*‐nitrophenolate (*p*‐NP) was plotted for each well and the rate of *p*‐NP accumulation was derived by calculating the slope at the linear phase of each reaction for each sample. The absorbance at 405 nm was measured by a benchtop microplate reader and the experiment was repeated three times and all samples were used in triplicates.

### 2.7 Real‐time reverse transcription polymerase chain reaction (qRT‐PCR)

Differentiation potential was examined using osteogenic markers osteopontin (OPN), RUNX family transcription factor 2 (RUNX2), osteocalcin (OCN), and the odontogenic marker dentin sialophosphoprotein (DSPP) using qRT‐PCR. hDPSCs (1 × 10^5^) were seeded in 6‐well plates and divided into five groups. After incubation for 2 weeks, the total RNA was extracted and reverse‐transcribed into complementary DNA with reverse transcriptase using the TaKaRa MiniBEST Universal RNA Extraction Kit and PrimeScript™ RT Master Mix (Perfect Real Time; Takara) according to the manufacturer's protocols. Subsequently, PCR amplification was performed with SYBR® Premix Ex Taq™ (Tli RNaseH Plus; Takara) using the ABI 7500 Thermal Cycler (Applied Biosystems). Relative gene expression was assessed using the $2^{-\mathrm{∆∆}C_{T}}$ method. As a negative control, cells cultured in the original complete medium were employed. Meanwhile, cells cultured in the osteogenic medium constituted the positive control group (referred to as the “osteogenic group”). The gene expression levels were normalized to the β‐actin messenger RNA (mRNA) level and averaged from triplicate samples. The data are expressed as means and standard deviations of three independent experiments performed in triplicate. The primer sequence of genes is shown in Table 1.

**Table 1:** Primer sequences of genes used in this study.

Gene	Forward primer (5′–3′)	Reverse primer (5′–3′)
*OPN*	CTAATTCAGAAAGGAAATGC	GCTGAGTGTTCTGGTGGACA
*RUNX2*	GTTATGAAAAACCAAGTAGCCAGGT	GTAATCTGACTCTGTCCTTGTGGAT
*OC*	CGCCTGGGTCTCTTCACTAC	CTCACACTCCTCGCCCTATT
*DSPP*	TCACAAGGGAGAAGGGAATG	TGCCATTTGCTGTGATGTTT

Abbreviations: DSPP, dentin sialophosphoprotein; OCN, osteocalcin; OPN, osteopontin; RUNX2, RUNX family transcription factor 2.

### 2.8 Statistical analysis

The results represent the means ± standard deviations and were derived from experiments performed in triplicate. The normality of the data was evaluated using the Shapiro–Wilk test. To ascertain the statistical significance among the experimental groups, a two‐way analysis of variance with Tukey's post hoc test was applied, considering a significance level of *p* < .05. The statistical analysis was conducted using GraphPad Prism 9.0 (GraphPad Software).

## 3 RESULTS

### 3.1 Stem cell isolation and culture

**Figure 1:**
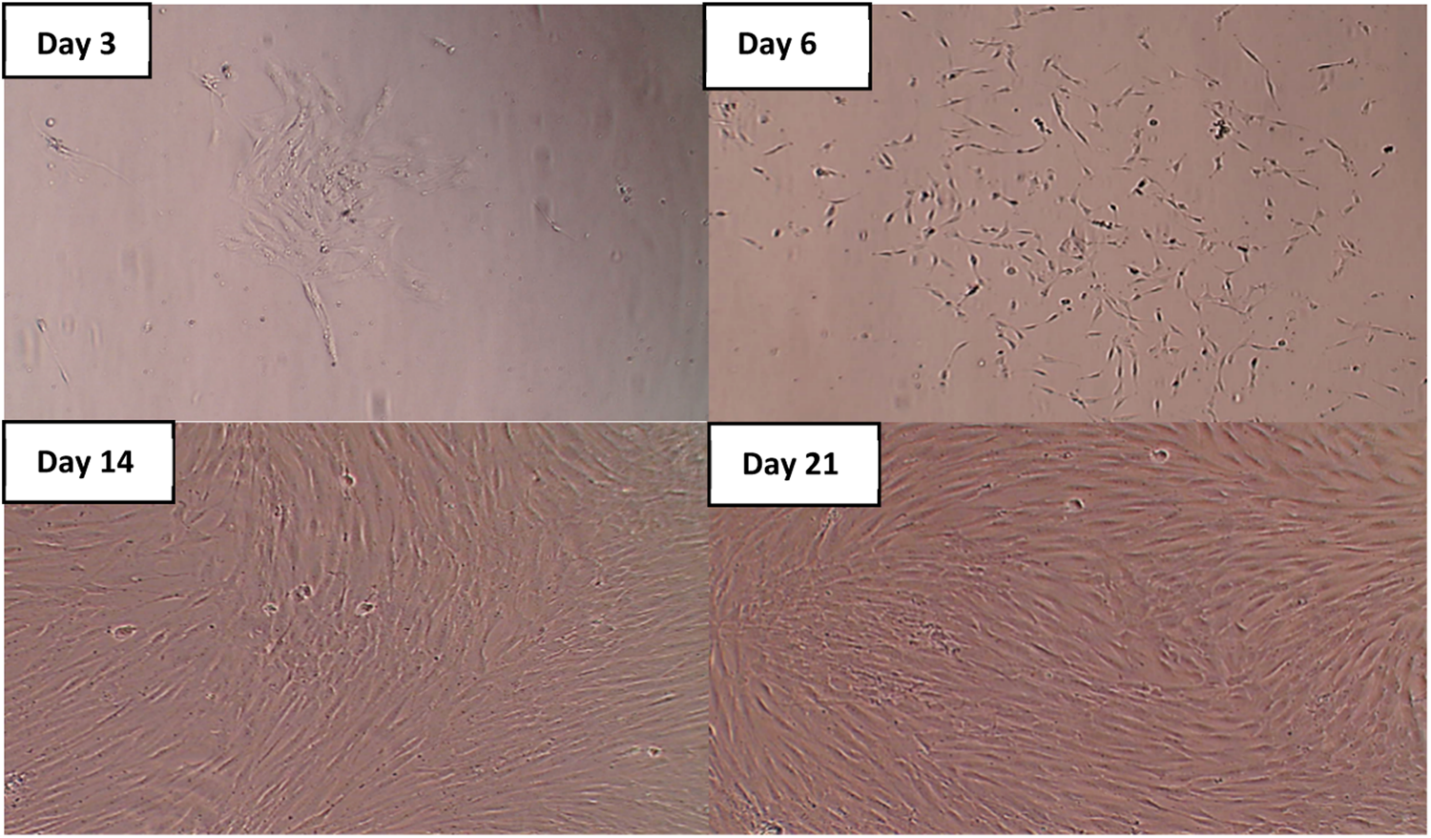
A photomicrograph showing dental pulp stem cells on Days 3, 6, 14, and 21.

hDPSCs were successfully isolated by enzymatic digestion and amplified by adherence separation, reaching 80% confluence by Day 14. Cells were observed using an inverted light microscope. The initial culture showed floating of small round cells, and by Day 7, the cells exhibited fibroblast‐like morphology. At Day 14, more cells exhibited spindle shape morphology and afterward there was increased cell proliferation reaching third passage by Day 21 (Figure 1).

### 3.2 Characterization

#### 3.2.1 Flow cytometry

**Figure 2:**
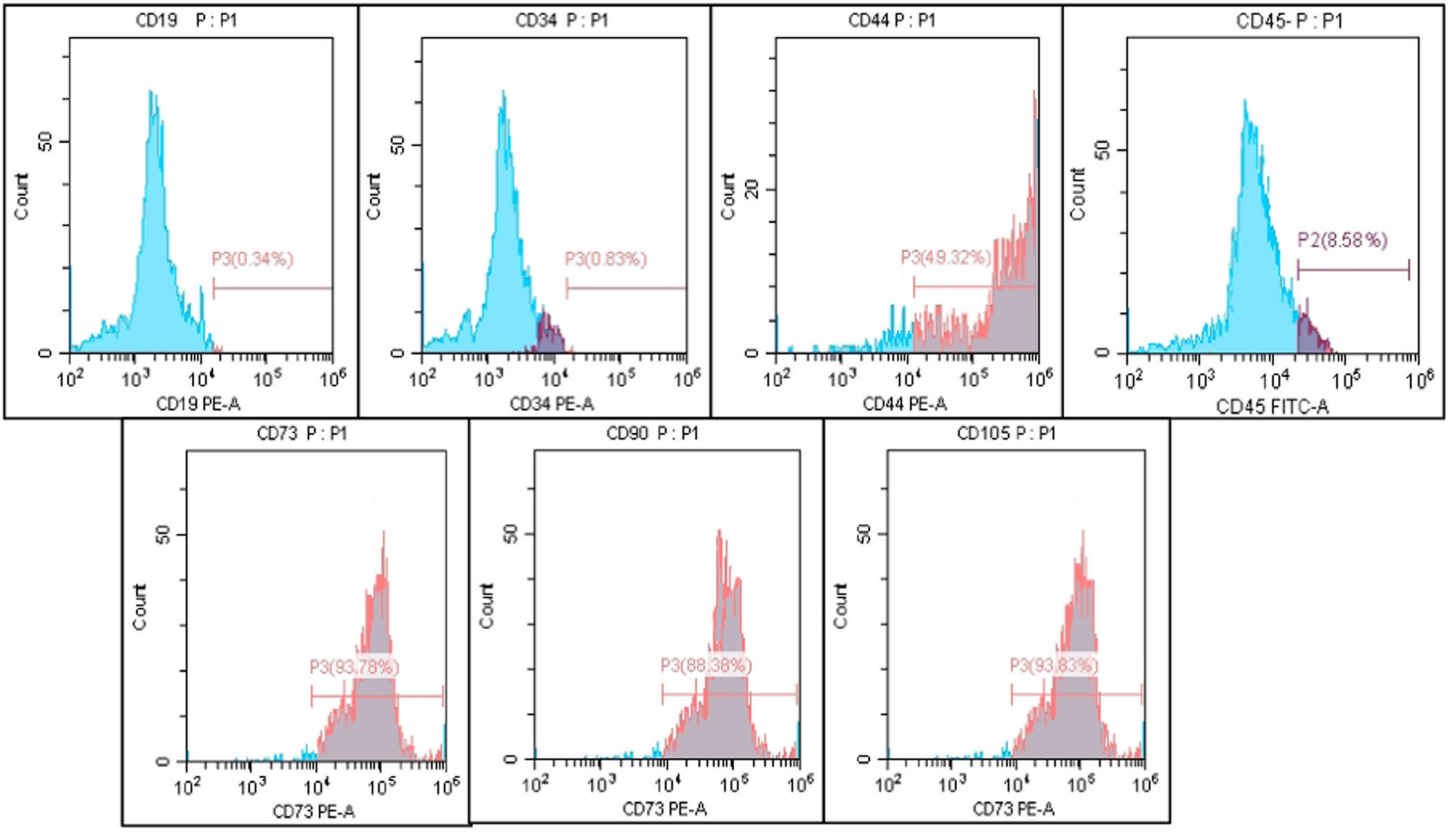
Surface marker expression by flow cytometry analysis.

To characterize the hDPSC population, surface markers were evaluated by flow cytometry. The third passage culture was used for immunophenotypic characterization. Results showed that hDPSCs were positive for MSC markers CD73, CD90, CD105, and CD44 but negative for hematopoietic stem cell markers CD45, CD34, and CD19 (Figure 2).

#### 3.2.2 Multilineage differentiation

**Figure 3:**
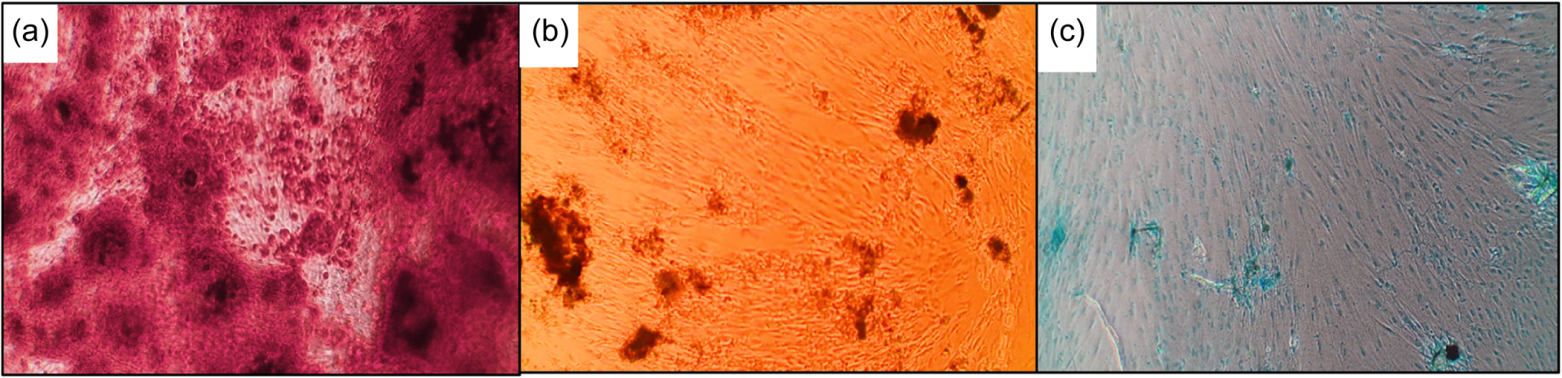
Human dental pulp stem cell differentiation into: (a) osteoblasts confirmed by Alizarin Red staining, (b) adipocytes confirmed by Oil Red O staining, and (c) chondrocytes confirmed by Alcian blue staining.

Trilineage mesenchymal differentiation of hDPSCs (osteogenic, adipogenic, and chondrogenic differentiation) was confirmed by morphological changes and special stains (Figure 3).

### 3.3 MTT assay (cell viability)

**Figure 4:**
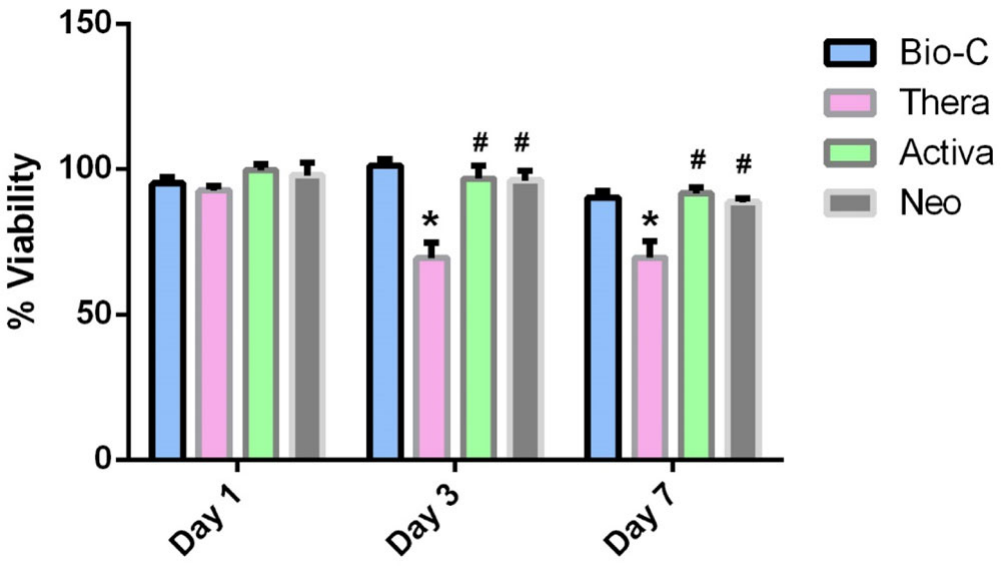
MTT (3‐(4,5‐dimethylthiazol‐2‐yl)‐2,5‐diphenyltetrazolium bromide) assay. Data are presented with absorbance values (570 nm) at Days 1, 3, and 7 of exposure of the hDPSCs to disc eluates. *p significant difference of the respective group compared to the Bio C group and ^#^ *p* significant difference of the respective group compared to the TheraCal group. *p* < .05 is statistically significant. Each experimental condition was performed in triplicate for each material's surface characteristics.

MTT assay was used to compare the cytotoxic effect of the dental materials on hDPSCs. The hDPSCs were incubated in α‐MEM growth medium containing 10% FBS in the presence of the tested materials. The cell viability of hDPSCs was measured on Days 1, 3, and 7 using MTT assay. The percentage of stem cell viability was compared to control (100%). Results showed that all capping materials showed variable cell viability against hDPSCs compared to control. hDPSCs of the Activa group showed 91.6% cell viability, followed by Bio C at 90.8%, followed by Neoputty at 88.5%, whereas TheraCal showed the least cell viability at 69.4% in comparison to control at Day 7. Multiple comparison test results are summarized in Figure 4.

### 3.4 Wound assay (cell migration)

**Figure 5:**
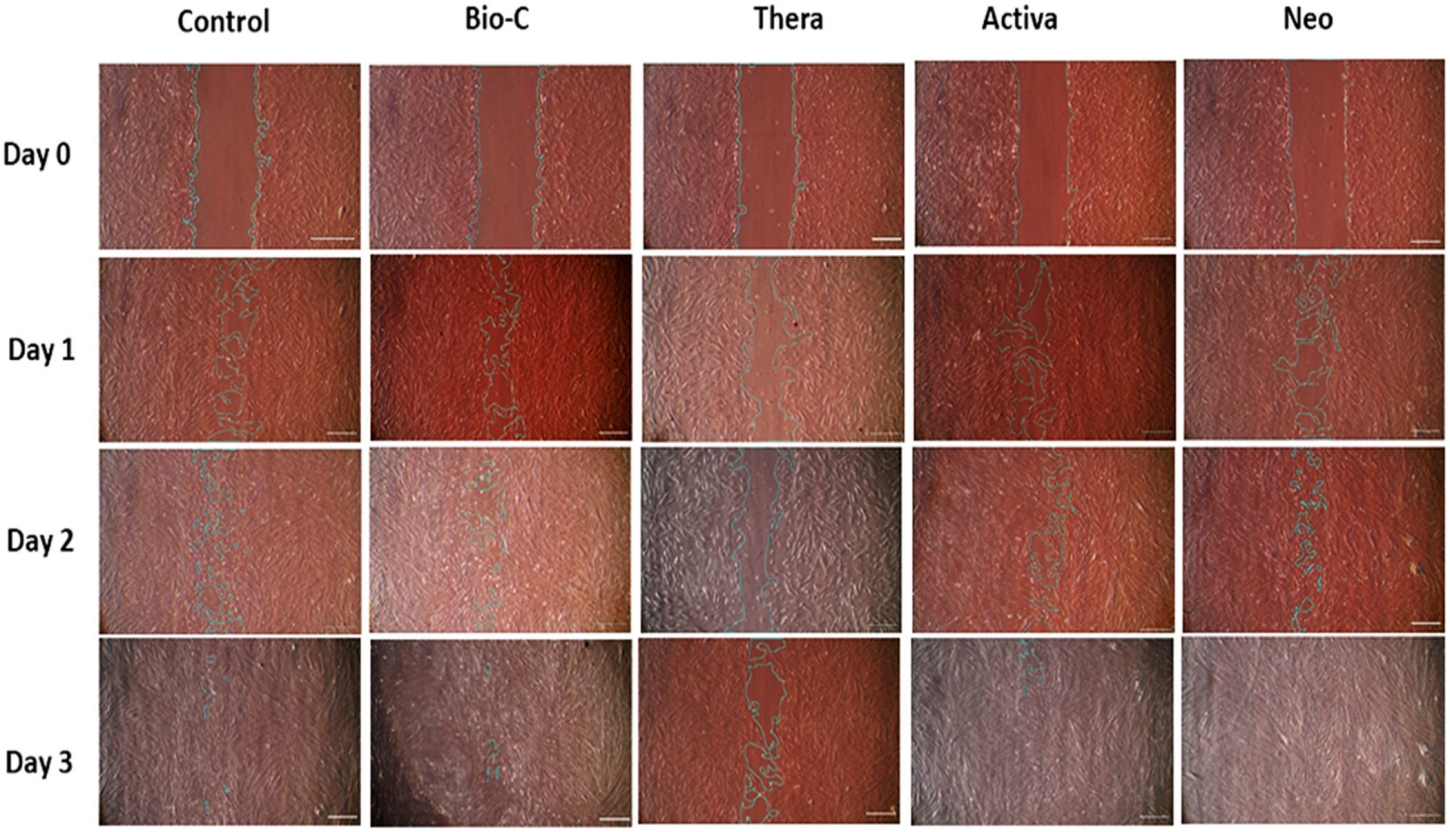
Wound closure at Days 0, 1, 2, and 3.

Cell migration assay was performed, and wound closure percentage was measured at Days 1, 2, and 3. At Day 3, Neoputty showed the highest wound closure percentage at 100%, followed by Bio C at 97.4%, and by Activa at 95.6%, whereas TheraCal revealed the lowest percentage at 56.2% (Figure 5).

**Figure 6:**
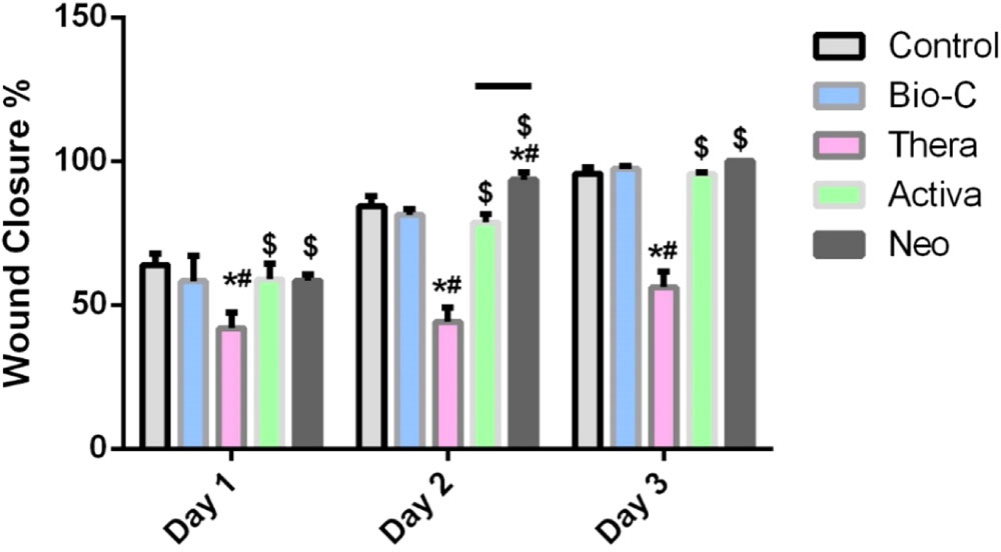
Wound closure percentage. The values reported are the means ± SD of Days 1, 2, and 3. *p < .05 represents a significant difference compared with the control group. ^#^ *p* significant difference of the respective group compared to the Bio‐C group. ^$^ *p* significant difference of the respective group compared to the TheraCal group.

Summary of mean and standard deviation of wound closure percentage and comparative statistical analysis are summarized in Figure 6.

### 3.5 Alizarin Red

On the 21st day from odontogenic differentiation, cultured cells were stained with Alizarin Red to identify nodules of calcification. After applying the osteogenic induction medium, the cells were observed regularly for morphological changes.

**Figure 7:**
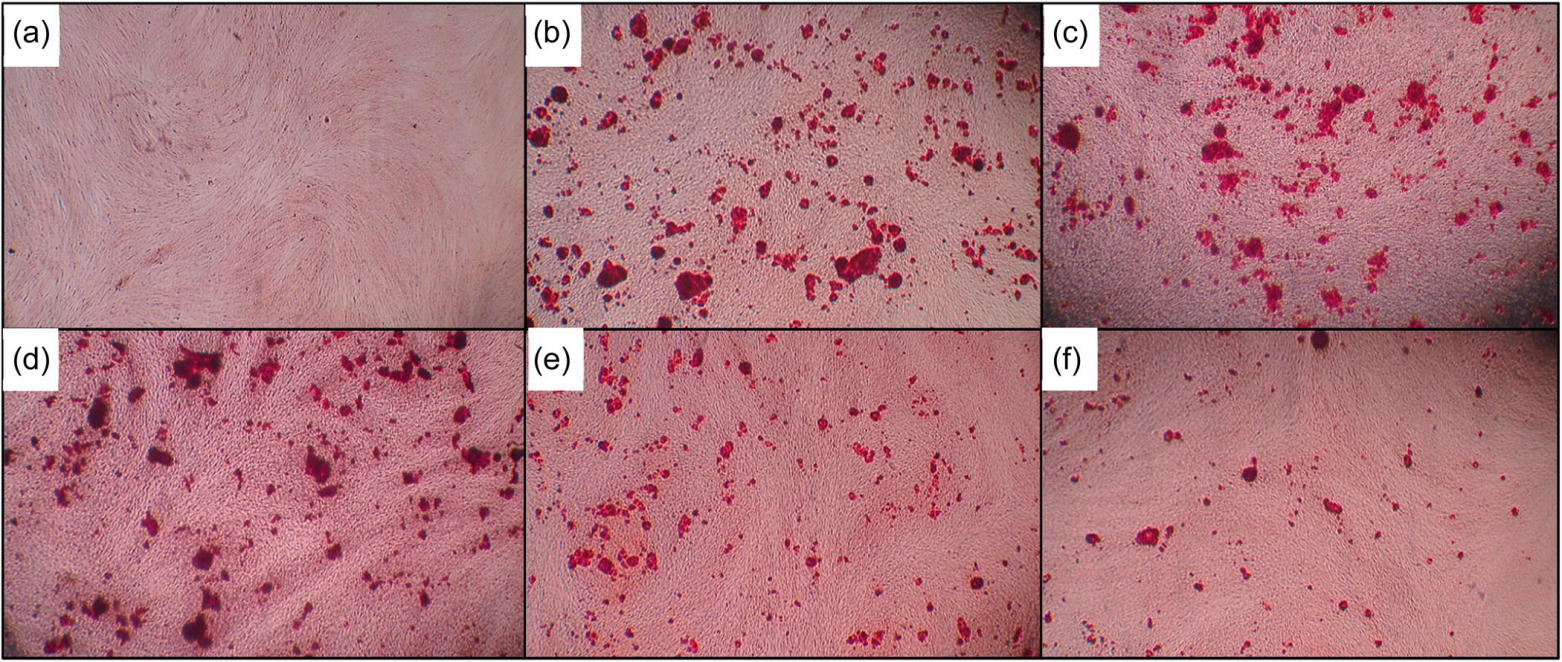
Odontogenic differentiation evaluated after 21 days using Alizarin Red staining of (a) Control, (b) odontogenic media, (c) Bio‐C, (d) Activa, (e) Neoputty, and (f) Theracal.

**Figure 8:**
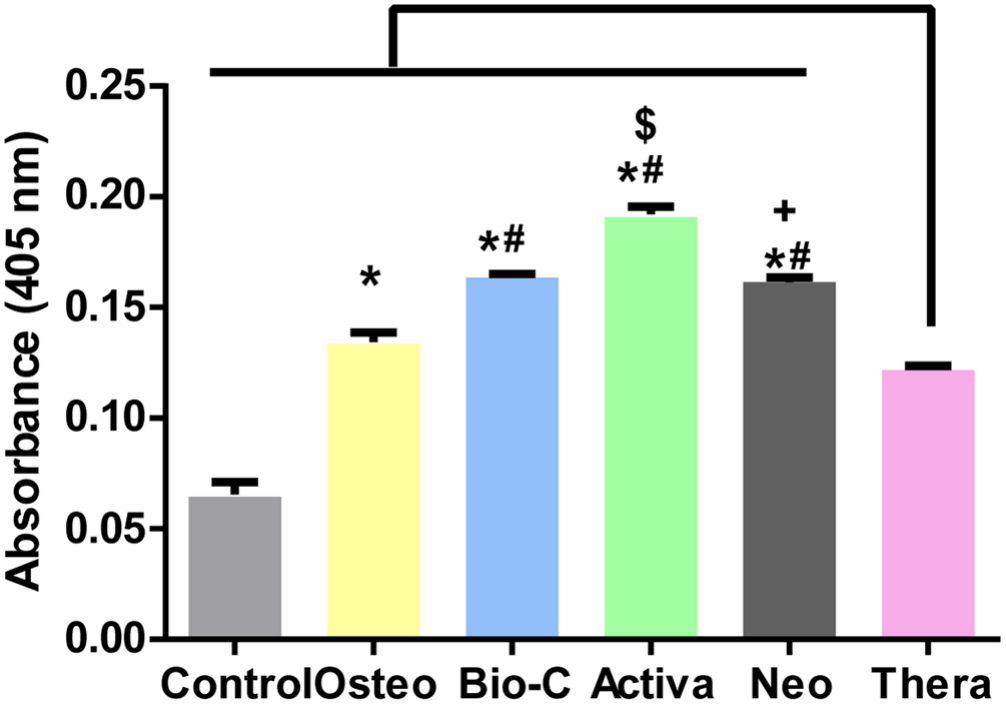
Quantitative measurements of Alizarin Red S staining of human dental pulp stem cells. The results are expressed as means and standard deviation and *p < .05 represents a significant difference compared with the control group. ^#^ *p* significant difference of the respective group compared to the TheraCal group. ^$^ *p* significant difference of the respective group compared to the osteogenic media group. ^+^ *p* significant difference of the respective group compared to the Activa group.

Alizarin Red stain was performed, and the aggregated cultured cells gave positive results indicating the formation of calcific deposits (Figure 7). By the 21st day, the staining became more intense and multiple isolated mineralized extracellular nodules appeared. The average absorbance rate for Alizarin Red staining of all groups was measured. Statistical analysis of the average absorbance rate is summarized in Figure 8.

### 3.6 ALP results

**Figure 9:**
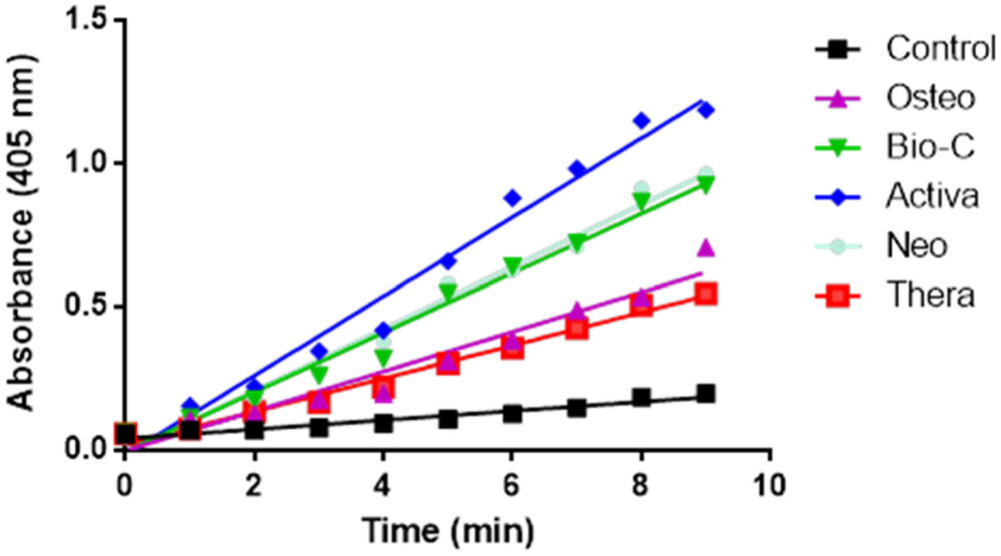
Representative kinetic profile of alkaline phosphatase assay demonstrating accumulation of the yellow *p*‐nitrophenolate product over time among the different groups.

The kinetic profile of alkaline phosphatase (ALP) assay demonstrating the accumulation of the yellow *p*‐NP product over time among the different groups is shown in Figure 9. The control group possessed the lowest rate of accumulation, while Activa group showed the highest rate.

**Figure 10:**
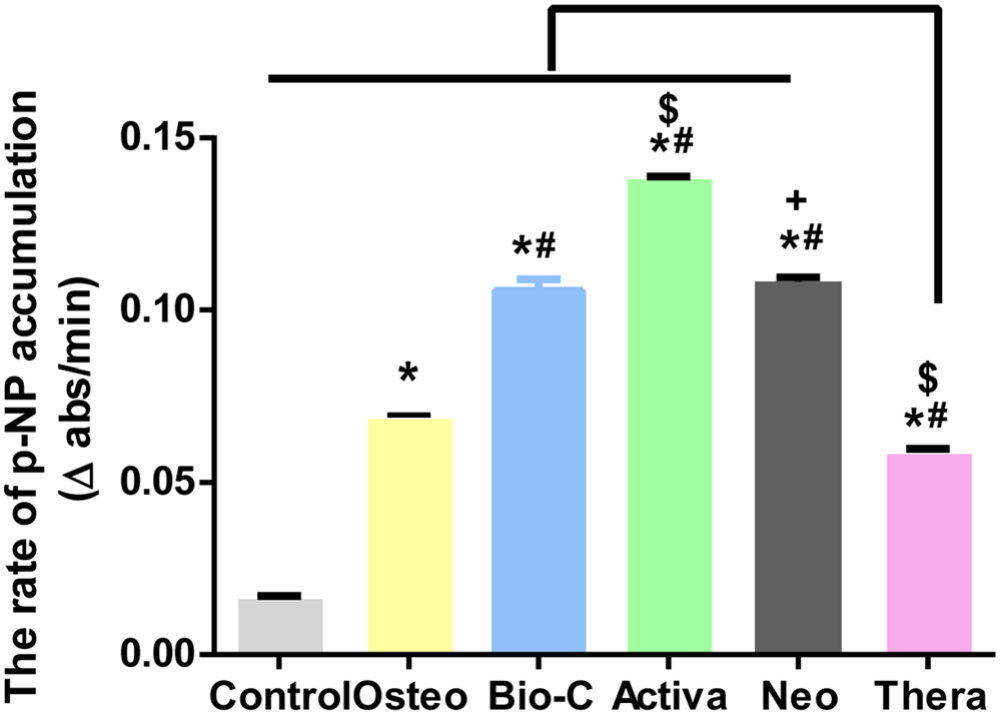
Rate of accumulation of the yellow *p*‐nitrophenolate (*p*‐NP) product. The values reported are the means ± SD of three independent experiments, *p significant difference of the respective group compared to the control (cells in normal medium), ^#^ *p* significant difference of the respective group compared to the osteogenic media, ^$^ *p* significant difference of the respective group compared to the Bio‐C group. ^+^ *p* significant difference of the respective group compared to the Activa group. *p* < .05 is statistically significant.

Statistical analysis was done by calculating the slope of each curve and dividing by the total amount of protein in each well. Summary of the means and standard deviations of the rate of *p*‐NP accumulation and statistical analysis are shown in Figure 10.

#### 3.6.1 PCR results

**Figure 11:**
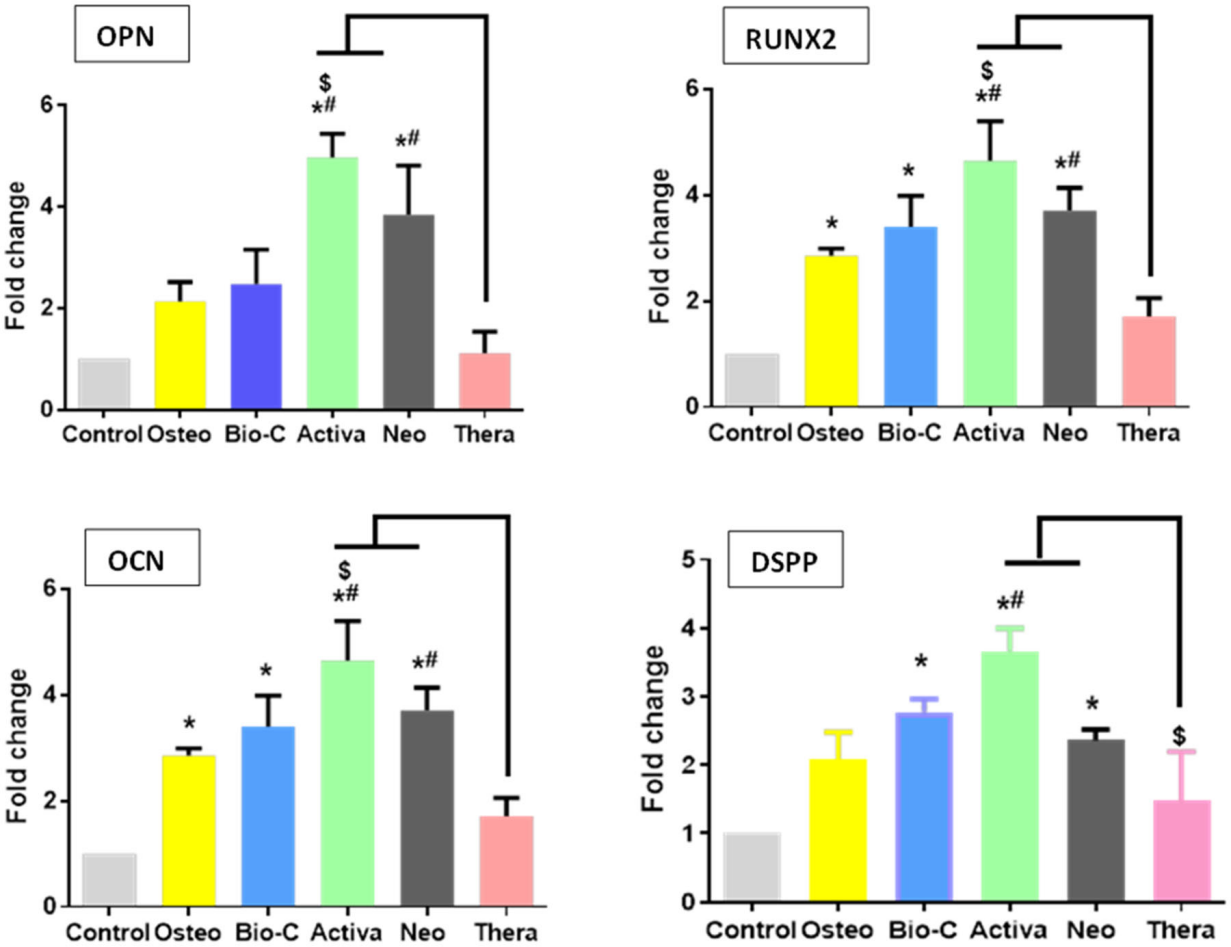
Effects of material extracts on the expression of osteogenic differentiation markers (OPN, RUNX2, OCN) and odontogenic marker (DSPP) in hDPSCs. Cells cultured with osteogenic differentiation medium were used as positive control group. The results are expressed as means and standard deviation and *p < .05 represents a significant difference compared with the control group. ^#^ *p* significant difference of the respective group compared to the TheraCal group. ^$^ *p* significant difference of the respective group compared to the osteogenic media group. DSPP, dentin sialophosphoprotein; hDPSC, human dental pulp stem cell; OCN, osteocalcin; OPN, osteopontin; RUNX2, RUNX family transcription factor 2.

Figure 11 summarizes the expression levels and statistical analysis of osteogenic (OPN, RUNX2, OCN) and odontogenic (DSPP) differentiation markers in hDPSCs for each group, which were normalized to β‐actin. Results showed that Activa exhibited significantly higher expression levels of OPN, RUNX2, OCN, and DSPP than control (*p* < .05). TheraCal revealed a nonsignificant expression of all genes in comparison to control (*p* > .05).

## 4 DISCUSSION

The odontogenic differentiation of hDPSCs has evolved over the years with multiple techniques being introduced for the purpose of creating a favorable environment for the attraction, adhesion, and multiplication of hDPSCs including the use of calcium phosphate porous granules (Nam et al., 2011) and calcium‐aluminate‐enriched chitosan–collagen scaffold as a potential candidate for developing an acellular means to dentin tissue engineering (Soares et al., 2017). Traditionally, pulp‐capping agents have been and are still being used for stimulation of the formation of a dentin bridge as well as for protection of the dentino‐pulp complex (Qureshi et al., 2014). The present study compared the effects of a number of vital pulp therapy materials using a number of variables to help inform clinicians about the advantages and strengths of each material which will help in decision‐making for a superior treatment outcome. With regard to hDPSC characterization, flow cytometry results showed positive expression of CD73, CD90, and CD105, while hematopoietic stem cell markers CD45, CD34, and CD19 were not expressed (Jun et al., 2017). The culture medium is an important factor in the rate of odontogenic differentiation of hDPSCs where induced DPSCs have a higher odontogenic differentiation potential than uninduced DPSCs and/or DPSCs cultured with differentiation medium only (Mohamed & Fayyad, 2017). In the present study, hDPSCs were characterized by flow cytometry analysis and multiple lineage differentiation potential into odontogenic, adipogenic, and chondrogenic cell lines.

The selection of materials tested in the current study was done carefully during the study design to represent different categories of pulp capping materials that have not been tested/compared against each other. Neoputty‐ MTA® is a relatively new premixed tricalcium silicate‐based material. To our knowledge, it has not been previously tested for odontogenic differentiation. Bio‐C sealer® is a new silicate‐based bioceramic cement that is presented in a ready‐for‐use format, and to our knowledge has not been tested for odontogenic differentiation of hDPSCs. Activa Bioactive® is a bioactive light‐cured resin‐modified glass ionomer cement that has been well‐established in literature as a reliable and promising pulp‐capping agent (Jun et al., 2017; Kunert & Lukomska‐Szymanska, 2020; Ranjbar Omrani et al., 2021). It should be noted that Theracal PT® is another relatively new dual‐cured resin‐modified calcium silicate‐based material that is different from TheraCal LC®. It has been tested in a few studies for cytocompatibility, bioactive properties (Rodríguez‐Lozano et al., 2021), and mineralization (Sanz et al., 2021), but not odontogenic differentiation; however, there has been plethora of studies that investigated different properties of TheraCal LC® (Bakhtiar et al., 2017; Farsi et al., 2018; Kang, 2020; Manaspon et al., 2021; Omidi et al., 2020). This study is the first to compare the above‐mentioned four materials with regard to biocompatibility and odontogenic differentiation with hDPSCs.

The current study is highly important in filling gaps within the available literature regards vital pulp therapy materials. A recently published literature review concluded that the evidence available was not sufficient to recommend the use of TheraCal LC® or Activa Bioactive® base/liner in vital pulp therapy (Kunert & Lukomska‐Szymanska, 2020; Omidi et al., 2020). Our results demonstrate that Activa Bioactive® base/liner is superior compared to TheraCal PT® in terms of cell viability, producing higher odontogenic differentiation, calcification nodules, ALP activity, and mRNA expression. A recently published case report confirmed the results of the present study (Mazumdar et al., 2020), which demonstrated that Activa Bioactive® base/liner showed promising results in the preservation of the pulp vitality and disappearance of pulpitis symptoms after a 1‐year follow‐up. This could be attributed to a better marginal adaptation of Activa Bioactive®, which might be superior to the less reliable marginal adaptation of MTA‐based dental pulp capping materials that have been previously reported (Sharaan et al., 2021). The significance of the current study is to allow for future animal experiments and clinical trials with Activa Bioactive® base/liner. Furthermore, another study (Jun et al., 2017) concluded that further in vivo studies at a gene/protein level are needed to investigate the releasable cytotoxic inducers during the light‐curing process to comprehend the cytotoxicity of BA and its effect on clinical treatment outcomes. The current study confirmed that Activa Bioactive® base/liner is a highly biocompatible material. It should be noted that the concentration of the diluted extract of tested materials could vary between studies (Jun et al., 2017).

It is intriguing that the formation of dentin bridge could be achieved through different mechanisms/pathways, for example, Ca(OH)_2_, MTA, and Biodentine™ stimulate cyclin D1 expression, a regulator of oral dysplasia (Bakr et al., 2018), while Biodentine^TM^ demonstrated a unique Wnt/β‐catenin signaling mechanism to promote dentin bridge formation (Yaemkleebbua et al., 2019). The picture gets more complicated with other factors being involved in a cascade of inflammatory/reparative mechanisms. A recent study (Peng et al., 2022) illustrated that silicate bioceramic‐based materials are responsible for the increased secretion of cytokines including fibroblast growth factor‐2 and tumor growth factor‐β1, which are important for odontoblast migration and differentiation. Furthermore, R‐Spondin 2 (Rspo2), which is a growth factor that is specific to stem cells, has been proven to promote the proliferation and odontogenic differentiation of hDPSCs by regulating the Wnt/β‐catenin signaling pathway (Gong et al., 2020). Finally, the microenvironment surrounding the odontogenic MSCs is very important in determining the outcome/prognosis of the pulpal regeneration process (Huang et al., 2021). Therefore, every effort should be made to simulate the pulp microenvironment in research settings.

Previous studies showed that MTA did not significantly alter ALP activity and calcium and mRNA expression levels of the dental pulp cells when compared to the negative group that received no treatment (Onay et al., 2018). This is contrary to the results obtained from this study and could be explained due to the difference in methodology between both studies where the other study used transwell barriers that could have blocked the activation of the dental pulp cells (Paranjpe et al., 2011). Our study design allowed indirect contact between the tested materials and hDPSCs through dilute extract, which validates the reliability of the current study and the close proximity of the research conditions to a real clinical scenario. Furthermore, the difference in the commercial MTA material used (Proroot MTA; Dentsply Tulsa) versus Neoputty MTA® in the current study. It is also important to note that a balance between the inflammatory response and the regenerative potential of the pulp capping material(s) should be taken into consideration (Giraud et al., 2019). It has been proven that TheraCal LC® shifts the balance toward an intense inflammatory reaction that could alter the regeneration process due to incomplete polymerization and/or hydration and decreased cell viability from the uncured monomers, as well as the release of cytokines (Bakhtiar et al., 2017; Jeanneau et al., 2017), while resin‐free materials induced regeneration with a subtle anti‐inflammatory reaction that inhibits proinflammatory factors and minimizes inflammatory cells recruitment, which results in the overall shift of the balance toward regeneration without compromising the healing process (Giraud et al., 2018; Nowicka et al., 2013). All of the above supports the findings from the current study where all test materials outperformed TheraCal PT®. Decreased cell viability does not necessarily indicate that a material is toxic. Therefore, the above results warrant the need for further testing of different materials' cytotoxicity using live‐dead staining. Furthermore, despite containing resin, Activa Bioactive® outperformed all test materials in the current study, which is explained by the nature of the resin component in Activa Bioactive®, an ionic resin matrix, as well as a shock‐absorbing resin where both layers closely resemble the physical and chemical properties of tooth structure to the extent that it has been used in the manufacturing of CAD/CAM blocks for monolithic crowns in the premolar area (Abdulla & Majeed, 2019). When the above is combined with the release of calcium, phosphate, and fluoride ions (May & Donly, 2017) that contributed to the higher ALP activity, calcification, and wound closure in the present study, it can be proposed that Activa Bioactive® combines the benefits of fracture resistance, biocompatibility, anti‐inflammatory, and regenerative properties.

Neoputty MTA® showed comparable results to Activa Bioactive® and Bio‐C sealer® in the present study. This is consistent with another study (Sun et al., 2017) where iRoot FS, a bioactive ceramic, and Biodentine™, a tricalcium silicate were compared and showed similar results; however, the results were variable according to the thickness of remaining dentin (Javid et al., 2020) as well as being concentration/dose‐dependent (Ali et al., 2021). Therefore, it is important as a future recommendation to trial different concentrations of the commercially available materials under different dentin thicknesses to optimize the results and provide tailor‐made recommendations to clinicians that will help inform the decision‐making process in different clinical scenarios.

TheraCal PT® was the least performing tested material in the present study and is in alignment with previous studies, which proved MTA and Biodentine™ had superior regenerative powers when compared to TheraCal LC® (Farsi et al., 2018; Kang, 2020; Manaspon et al., 2021; Omidi et al., 2020). This was further supported by Babaki et al. (2020) who concluded that MTA is promising with regard to stem‐cell‐based endodontic approaches due to its osteo/odontogenic differentiation‐inducing properties, but further animal/human trials were deemed necessary. Despite TheraCal PT® showing superior properties to TheraCal LC® (Sanz et al., 2021), it seems that the TheraCal family of pulp‐capping agents failed to outperform other materials as shown in the present study.

The developments, innovations, and new trends in pulp regeneration techniques and materials will continue to rapidly evolve. An example of recent advancements include the potential use of aspirin in the odontogenesis of hDPSCs (Khampatee et al., 2022), the trial of fluocinolone acetonide as a pulp‐capping agent due to its anti‐inflammatory properties (Louwakul et al., 2021), an experimental pulp capping material composed of an antibacterial resin monomer (2‐methacryloxylethyl dodecyl methyl ammonium bromide, MAE‐DB) and Portland cement was investigated (Yu et al., 2016), the possibility of reparative dentin formation and hDPSC proliferation after the application of strontium‐substituted tetracalcium phosphate cement (Basheer 2021), the potential role of magnesium oxine in dentinogensis (Salem et al., 2021), the investigation of Rspo2 in the regulation of the Wnt/β‐catenin signaling pathway (Gong et al., 2020), the role of mesoporous bioactive glass in odontogenic differentiation of hDPSCs (Zhu et al., 2021), the possible functions of icariin in odontogenic differentiation of hDPSCs by triggering the mitogen‐activated protein kinase signaling pathway (Liu et al., 2022), and the investigation of the effect of *Moringa oleifera* on hDPSCs (Salem et al., 2022). In addition to that, enamel matrix derivatives have been investigated for pulpal regeneration due to their ability to stimulate the proliferation of odontoblast/osteoblast‐like cells for a long time (Dahake et al., 2020; Najeeb et al., 2017; Nakamura et al., 2001; Wang et al., 2014; Youssef et al., 2019). The results demonstrated the potential for enhanced mineralization and expression of dentine sialoprotein (Dahake et al., 2020). The investigations will still continue due to the need for long‐term follow‐up data (Najeeb et al., 2017). Finally, platelet‑rich fibrin and concentrated growth factors have been investigated in in vitro studies, which showed promising results in reducing the apoptotic rate and increasing the viability and proliferation rate of the human dental pulp when compared to Ca(OH)_2_ and MTA (Dou et al., 2020).

The findings from the present study support further exploration of the use of resin‐modified glass ionomers and suggest the potential need to shift from calcium silicates to new alternatives, especially with the current developments in bioceramics and biological scaffolds (Cassiano et al., 2020; Hanna et al., 2020). The findings from the current study also support the idea that a one‐product‐fits‐all approach does not exist and further studies are needed to recommend specific bioceramic products to suit different clinical scenarios such as trauma versus caries or direct versus indirect pulp capping (Ali et al., 2021). It seems to be the case that regenerative medicine has progressed rapidly when compared to regenerative dentistry; therefore, further in vitro, animal studies and clinical trials are required for long‐term follow‐up data (Whitehouse et al., 2021; Xiao & Nasu, 2014).

## 5 CONCLUSIONS

The results of this study were inconclusive regards contemporary bioceramic materials designed for vital pulp therapy as they have different effects on hDPSC. Further testing for cytotoxicity using live‐dead staining, animal experiments, clinical trials, and independent analyses of these biomaterials is necessary for clinicians to make an informed decision for their use.

## AUTHOR CONTRIBUTIONS

*Study conceptualization*: Mahmoud M. Bakr and Mohamed Shamel. *Data handling*: Mohamed Shamel, Shereen N. Raafat, and Mahmoud M. Al‐Ankily. *Experimental design*: Mahmoud M. Bakr, Mohamed Shamel, and Robert M. Love. *Analysis and interpretation of data*: Mahmoud M. Bakr, Mohamed Shamel, and Robert M Love. *Provision of study materials and equipment*: Mahmoud M. Bakr, Mohamed Shamel, and Robert M. Love. *Study validation and data presentation*: Mahmoud M. Al‐Ankily and Shereen N. Raafat. *Supervision*: Mahmoud M. Bakr and Robert M. Love. *Draft preparation*: Mahmoud M. Bakr, Mohamed Shamel, and Mahmoud M. Al‐Ankily. *Study consultation*: Mahmoud M. Bakr, Shereen N. Raafat, and Robert M. Love. *Writing and reviewing and project administration*: Mahmoud M. Bakr, Mahmoud M. Al‐Ankily, and Shereen N. Raafat. All authors approved the final version of this article.

## CONFLICT OF INTEREST STATEMENT

The authors declare no conflict of interest.

## ETHICS STATEMENT

The study protocol was approved by the Research Ethics Committee of the Faculty of Dentistry, The British University in Egypt (22‐008). All experimental procedures were performed in the Centre of Innovative Dental Science (CIDS) at The British University in Egypt.

## Data Availability

The data that support the findings of this study are available from the corresponding author upon reasonable request. The project data can be made available upon request from the corresponding author.
